# Strain Echocardiography in Acute COVID-19 and Post-COVID Syndrome: More than Just a Snapshot

**DOI:** 10.3390/biomedicines11041236

**Published:** 2023-04-21

**Authors:** Johannes Kersten, Jana Schellenberg, Achim Jerg, Johannes Kirsten, Hasema Persch, Yuefei Liu, Jürgen M. Steinacker

**Affiliations:** Division for Sports and Rehabilitation Medicine, University Hospital of Ulm, 89075 Ulm, Germany

**Keywords:** COVID-19, strain, post-COVID, speckle-tracking echocardiography

## Abstract

Speckle-tracking echocardiography (STE) has become an established, widely available diagnostic method in the past few years, making its value clear in cases of COVID-19 and the further course of the disease, including post-COVID syndrome. Since the beginning of the pandemic, many studies have been published on the use of STE in this condition, enabling, on the one hand, a better understanding of myocardial involvement in COVID-19 and, on the other, a better identification of risk to patients, although some questions remain unanswered in regard to specific pathomechanisms, especially in post-COVID patients. This review takes a closer look at current findings and potential future developments by summarising the extant data on the use of STE, with a focus on left and right ventricular longitudinal strain.

## 1. Introduction

**Figure 1:**
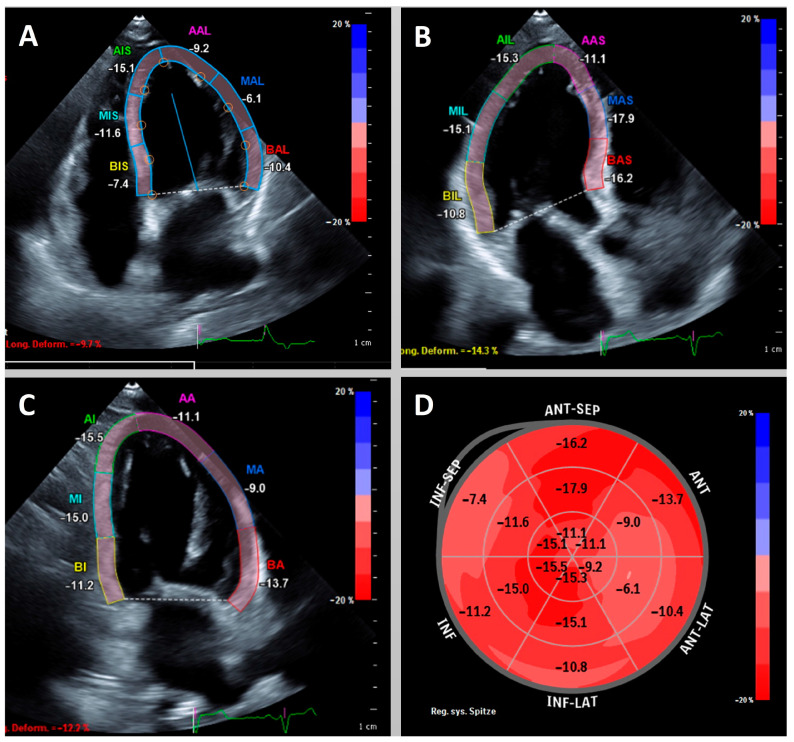
Example of a patient with reduced left ventricular global longitudinal strain (GLS) of −12.1%. Apical 4-, 3- and 2-chamber view (**A**–**C**) with highlighted left ventricular myocardium. Bullseye plot (**D**) shows globally reduced longitudinal strain with accentuation basally septal and midventricular anterior/anterolateral.

**Figure 2:**
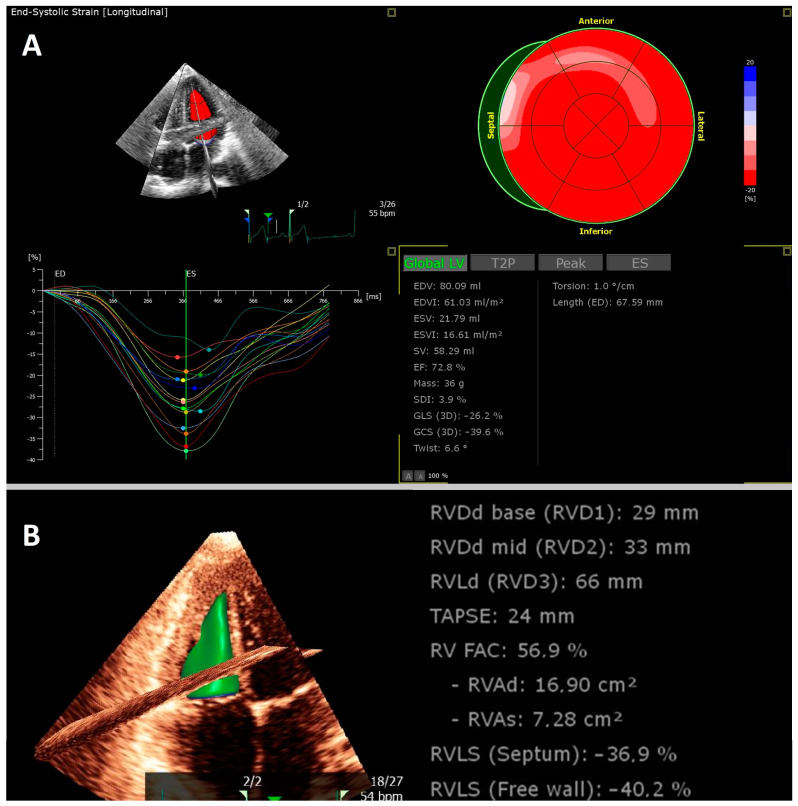
Example of a young athlete who was examined after COVID-19 before returning to sport. Transthoracic 3D echocardiography revealed normal left ventricular (**A**) and right ventricular function (**B**).

Speckle-tracking echocardiography (STE), which in daily practice most commonly measures left and right ventricular global longitudinal strain (LV GLS and RV GLS, respectively), is increasingly used to evaluate myocardial function, and this assessment is now considered a valuable tool in the diagnosis and management of all types of cardiomyopathies. Strain, defined as the percentage change in the length of the myocardium, provides a measure of the myocardium’s deformation in response to changes in pressure or volume. Strain values can be derived in each direction (longitudinal, radial, circumferential) and may be assessed from 2D and 3D images as well as in post-processing. STE has proven its value in diverse cardiomyopathies, such as takotsubo cardiomyopathy, myocarditis, cancer therapy related cardiac dysfunction, coronary artery disease and myocardial infarction [1,2,3,4,5]. An example of a reduced LV GLS using 2D-STE is shown in Figure 1, and an example of 3D-STE in a healthy athlete is shown in Figure 2.

Using STE in SARS-CoV-2 virus (COVID-19) patients made sense, as myocardial involvement was commonly found in these infections, especially in critically ill patients. Even in mild forms of myocardial dysfunction, deformation parameters are more accurate than classical volumetric evaluation and ejection fractions and have demonstrated good inter- and intra-observer reliability. Furthermore, the ability to perform echocardiography even at the bedside in a hospital ward gives STE an advantage over other imaging modalities, such as cardiovascular magnetic resonance (CMR) imaging and cardiac computed tomography.

In acute COVID-19, myocardial involvement has been shown to be prognostically significant, with increased mortality in patients with elevated troponin [6]. Various possibilities of myocardial involvement may be conceived, and myocardial infarctions, myocarditis and micro- and macro-embolisms have been detected in autopsy studies [7]. Arrhythmias and stress cardiomyopathies have also been described and affect patient prognosis [8,9]. The pathogenesis is diverse including viral endothelitis, humoral and prothrombotic changes and systemic inflammatory response syndrome [10,11,12]. In particular, the co-expression of angiotensin convertase enzyme 2 (ACE-2) and transmembrane serine protease 2 (TMPRSS2) on the cell surfaces of some tissues as the site of invasion of SARS-CoV-2 appears to have an impact on disease progression and multi-organ involvement [13,14].

Myocardial changes have also been reported in the post-COVID setting. *Post-COVID* describes a syndrome emerging in the context of COVID-19 that involves persistent symptoms even after three or more months, including fatigue, dyspnoea and chest pain [15,16]. Some studies have identified the presence of heart failure, myocarditis and left ventricular dysfunction following COVID-19, but further research is needed to fully understand the long-term cardiac impact of COVID-19 and the mechanisms underlying these changes. A loss of myocardial function is often mild and observable only in deformation parameters, not in common volumetric assessments, such as the left ventricular ejection fraction.

This review describes the current state of the use of STE in the acute COVID-19 and post-COVID contexts. We illustrate the potential and limitations of the methodology and highlight avenues for further development.

## 2. Strain in Acute COVID-19

The COVID-19 pandemic demanded a high degree of flexibility from the medical system. Especially in severely ill patients in the alpha- and delta-dominant waves, myocardial involvement was frequently observed with a poor outcome. Hygiene measures were adopted to provide the highest level of safety for medical staffs, but, in many hospitals, this limited the availability of diagnostic measures, e.g., CT and MRI imaging. Consequently, the possibility was soon considered of conducting bedside examinations using STE. An early article by Stöbe et al., which described a comprehensive examination protocol with LV strain in all three orientations in 19 patients during the first wave of the disease, hypothesised that STE could diagnose the myocardial involvement of COVID-19 [17]. A pattern of myocardial dysfunction was seen, with changes predominantly in the inferolateral and anterolateral basal segments. Free wall RV LS was reduced in 4 of the 19 patients and was accompanied by elevation of troponin T and NT-proBNP.

In a small prospective study of only 30 patients with signs of myocardial involvement marked by elevated troponin I, a significantly reduced free wall RV LS was found in nonsurvivors as compared to survivors (−15.6% vs. −24.3%, respectively, *p* = 0.0018) [18]. Li et al. further investigated the observed impairments in right ventricular strain [19]. In their single-centre study, patients with COVID-19 were examined bedside in their wards on the seventh day (on average) after hospital admission. Patients with known ischaemic and nonischaemic cardiomyopathy were excluded. The final 120 evaluable patients were divided into tertiles according to their free wall RV LS. Patients in the lowest tertile (−10.3% to −20.5%) had acute heart injury, acute respiratory distress syndrome (ARDS) and deep vein thrombosis more often than those in the other tertiles, and the latter finding especially suggests the possibility of thromboembolic events. A cut-off value of worse than −23% in free wall RV LS was associated with a significantly increased mortality. In a receiver operating characteristics analysis, the area under the curve (AUC) of free wall RV LS was 0.87 and thus clearly superior to the fractional area change (FAC) and the tricuspid annular plane systolic excursion (TAPSE) (which yielded AUC 0.72 and AUC 0.67, respectively).

A later study by Bleakley et al. of 90 critically ill patients requiring venovenous extracorporeal membrane oxygenation (ECMO) also examined the parameters of right ventricular function [20]. Classical parameters, such as diameter and FAC, which assess radial rather than longitudinal function, were significantly more likely to diagnose right ventricular dysfunction than free wall RV LS. The authors discuss an altered pattern of right ventricular dysfunction due to the more acute right ventricular afterload increase caused by endotheliitis, microembolism and macroembolism in the pulmonary stromal bed, with different influences on the right ventricular microstructure than those of chronic stress. Whether the specific setting of ECMO patients has an influence remains unclear.

Other studies examining right ventricular strain produced diverse results. For example, free wall RV LS was an independent predictor of mortality in a study of 132 patients by Xie et al., while Park et al. found no association with that outcome in only 48 patients [21,22]. Both studies took place in a similar time frame, so a different SARS-CoV-2 variant could not have significantly influenced the results.

A significant reduction of LV GLS has also been observed in COVID-19 patients. The degree of impairment correlated with NT-proBNP, troponin and inflammatory parameters in several studies [23,24], and an impaired LV GLS was associated with higher mortality. A correlation to the specific SARS-CoV-2 variant was not detectable in the influence on deformation parameters. In a study by Ghantous et al. of 148 patients infected with the omicron variant, no significant differences were found in LV GLS, RV GLS and free wall RV LS in comparison to a matched cohort with the wild-type variant [25], but differences in right ventricular geometry (FAC, RV end-systolic area) and maximal tricuspid regurgitation velocity were found in favour of the omicron-infected patients. In addition, the authors found lower values for the echocardiographically estimated pulmonary vascular resistance index, which they attribute to less lung parenchymal or vascular damage, fewer cytokine storms or combined effects. A study by Bhatia et al. also examined LV GLS in patients with COVID-19 [26]. They found reduced LV GLS values in 91% of patients, with a median value of −13.5% [IQR −15.0%, −10.8%] despite a normal left ventricular ejection fraction (LVEF) with a median of 62% [IQR 56%, 68%]. In this small cohort with follow-up examinations during inpatient stay, there was a trend towards improvement in the LV GLS in patients who could be discharged in contrast to patients with fatal courses.

Strain analysis of the atrial function is also possible, even if most examiners have not yet incorporated it into their daily routine. In a study of left atrial strain in critically ill patients requiring intensive care, left atrial strain was better able to identify patients with diastolic dysfunction than classical echocardiographic parameters [27]. Impaired left atrial strain was correlated with protracted inflammatory states and changes in the differential blood count.

D’Andrea et al. studied 55 patients with COVID-19-associated CMR-diagnosed myocarditis according to the updated Lake Louise criteria [28]. The CMR occurred during the first 14 days after hospital admission. Compared to matched healthy controls, the myocarditis patients saw a reduction in LV GLS, which was correlated to absolute scar burden in terms of late gadolinium enhancement. A follow-up examination showed a functional improvement in the myocarditis patients. Baseline LV GLS, LVEF and the extent of late gadolinium enhancement were independent predictors of LVEF at six months, which leads to the next section. (Table 1 summarises current studies of STE in COVID-19 patients.)

## 3. Strain in the Diagnostic Work-Up of Post-COVID Syndrome

A strength of STE is that it detects discrete, subclinical functional changes. The published data mostly describe only minor changes that do not reach pathological values in post-COVID patients. However, discrete impairments can be detected in comparison to healthy controls [29,30,31,32]. When interpreting the results of the following studies, it is partly necessary to take a close look at the inclusion criteria. A detailed presentation of these would go beyond the limits of this review due to the heterogeneity and their complexity.

A study by Caiado et al. of 100 post-COVID patients with an examination after 130 ± 70 days found no significant differences in LV GLS compared to healthy subjects [33]. There was a local change in the function of basal segments, however, which is consistent with Stöbe et al.’s descriptions in acute disease as discussed above [17]. 

Actual epidemiological data are not yet available on the incidence of STE changes in post-COVID patients. Data from the Epidemiology of Long Covid (EPILOC) study will provide further information in the near future. A study (without STE) by Petersen et al. of 443 patients 9.6 months after COVID-19 found reduced LVEF and increased levels of troponin T [34]. With numerical differences of 1.17% and 0.17 ng/L, respectively, the influence on patient treatment is questionable despite their statistical significance. To our knowledge, the largest study to date on STE in post-COVID patients is that of Garcia-Zamora et al. [35], which included 595 patients referred for echocardiography after COVID-19 in 10 hospitals in South America. There was a reduction in LV GLS (defined as >−18.0%) in 5.7% of the patients and a reduction in RV GLS (defined as >−20.0%) in 3.0%.

A study by Young et al. found no significant changes in LV GLS, RV GLS or free wall RV LS among 259 patients on whom STE was performed both before and after COVID-19 [36]. Only 27 patients experienced a worsening of a strain parameter, while 49 patients experienced new or worsening symptoms in the context of COVID-19 (19%). Patients who exhibited the worsening of a strain parameter during COVID-19 were significantly clustered in the group of patients with new symptoms.

Oikonomou and colleagues found a significant reduction in LV GLS in 34 post-COVID patients with STE compared to a healthy cohort (−18.4% vs. −22.0%, respectively, *p* < 0.001) [30]. LV GLS improved significantly in this COVID cohort up to the six-month follow-up but did not reach the levels seen in healthy subjects. In contrast, Baruch et al.’s study observed no improvement in LV GLS 88 days after infection. In that study, reduced LV GLS values were detected in 33% of patients during their hospitalisation (>−16.1% reduction for men and > −17.3% for women) [37]. At follow-up, this was the case in only 25%, which represents a numerical but not statistically significant improvement. A study by Lassen et al. also found no significant improvement in LV GLS [29]. The researchers studied 91 patients who had to be hospitalised because of COVID-19. They found a reduced LV GLS that did not improve in the follow-up examination 77 days (on average) after the first examination (−17.4 ± 2.9% vs. −17.6 ± 3.3%, respectively, *p* = 0.6). In contrast, there was a significant improvement in RV GLS (−25.3 ± 5.5% vs. −19.9 ± 5.8%, respectively, *p* < 0.001), in which the results are consistent with those of Baruch et al. [37].

Relevant long-term symptoms are often found even in mild courses of initial COVID-19. Especially in the case of dyspnoea, the question of myocardial damage thus also arises in oligosymptomatic courses. Indeed, Akkaya et al. found a relevant right ventricular dysfunction in their study of 105 mildly ill patients compared to a healthy control cohort [38]. Both RV GLS (−15.1 ± 3.4% vs. −19.6 ± 5.2%, *p* < 0.001) and free wall RV LS (−17.2 ± 4.4% vs. −19.6 ± 5.2%, *p* < 0.001) were significantly reduced in the post-COVID group as compared to controls. This study also found a correlation of the level of inflammatory blood values and D-dimers with right ventricular dysfunction. Therefore, thromboembolic and vasculitic mechanisms are also discussed in this patient population, which seems reasonable, as haematological, inflammatory and rheological changes occur under COVID-19 and often persist for a long time [11,39].

Other studies have found a clear correlation between initial disease severity and right ventricular function in STE during follow-up [32,40]. Hospitalised patients and patients with severe COVID-19 pneumonia were associated with reduced RV strain, and left ventricular function has exhibited similar correlations. For example, in a study by Mahajan et al., LV GLS was significantly worse in patients with severe COVID-19 than in those with moderate or mild disease severity (−15.5 ± 3.1% vs. −18.1 ± 6.9% vs. −21.0 ± 3.4%, *p* < 0.001) [41]. Overall, 29.9% had reduced LV GLS, which was defined as a value below the mean of a control group studied with the same protocol (−19.2%). This approach potentially overestimates the number of ‘pathological’ findings. The usual adjustment is ±1.96 standard deviations, and other authors obtain threshold values for LV GLS of −16.7% for men and −17.8% for women [42]; this adjustment would significantly reduce the number of abnormal findings in the study of Mahajan et al.

Most investigations find a normal ejection fraction with discrete changes in STE. In fact, the question arises of whether these echocardiographic changes are attributable to the often-high symptom burden or are merely an epiphenomenon. Several studies report correlations between an accumulation of abnormal STE values and patients with increased symptoms [36,43,44,45]. Because this is not consistently detectable in all symptomatic patients with post-COVID, however, a noncausal correlation must be assumed. Discretely elevated inflammatory biomarkers are detectable particularly in patients with a high symptom burden. Relatedly, in other conditions, myocardial dysfunction is often associated with an increase in these markers [46]. In this respect, a reduction of the deformation parameters in post-COVID could be a surrogate parameter of an ongoing disease process. However, other causes of functional abnormalities in patients with a higher symptom burden like detraining must be discussed, as well [47,48].

This assumption may also be supported by another study. In their study of 184 post-COVID patients, Shimoni et al. report a correlation of reduced maximal oxygen uptake in cardiopulmonary exercise testing with reduced LV GLS [31], which suggests a more systemic disease state. Regarding the right ventricle and its good regenerative capacity after acute COVID-19 as mentioned above, pulmonary changes are more likely (e.g., vascular scarification in the case of lung damage, thromboembolism).

In the work-up of patients with persistent symptoms after COVID-19, pre-existing conditions must be considered, as well [49]. These can also be unrecognised so far undetected, such as subclinical coronary artery disease, which also goes in line with a reduced global or regional strain [5]. In a study by Gherbesi et al., this confounder can be excluded [50]. Here, 40 young athletes aged 24.4 ± 8.3 years had worse LV GLS (but not RV GLS) than age- and gender-matched controls. The clinical impact in this asymptomatic cohort with a LV GLS of −22.7 ± 1.6% is unclear. Screening for myocardial involvement in asymptomatic patients is not recommended [49].

Of course, irreversible changes and damage in the context of acute COVID-19 may also be causal for the described changes in STE. For example, post-myocardial scars can lead to a regional restriction of the deformation parameters [28]. In their study of 123 patients, Italia et al. describe a significant reduction in LV GLS in patients with persistently elevated levels of troponin T [51], which suggests persistent and ongoing myocardial damage as the cause of myocardial dysfunction. Studies with cardiovascular magnetic resonance imaging suggest a complex picture with ischaemic and inflammatory signs in noninvasive tissue characterization [52,53]. The cause of such a persistent injury is ultimately unclear, and diverse hypotheses have been proposed, including immunological aberrations, autoimmunity, a change in the patient’s microbiome, metabolic dysregulation and microvascular, endothelial or hormonal dysfunctions [35,54,55,56]. Post-COVID is a multifactorial disease in which microvascular perfusion disturbances and inflammation may be most important denominators for cardiac dysfunction [49]. However, the pathophysiology should be eluded in cardiovascular studies which include parameters of microvascular perfusion and inflammation (Table 2 provides an overview of current data).

## 4. Current Limitations of Knowledge and Future Directions

The present review has attempted to give a comprehensive overview of the current data on STE in COVID-19 and post-COVID syndrome. This revealed a great heterogeneity of the studies. Disease severity of COVID-19 and time of the exams were very different, so that presumably no meta-analysis will be possible. Heterogeneity of the cohorts and often a small sample size are most likely the reasons for sometimes contradictory results.

It must also be borne in mind that STE itself has relevant limitations. There is a risk of foreshortening and through-plane motion [57]. There is also a certain dependence of the STE measurement on the vendor and the software used [42,57,58]. Due to a high dependence on image quality, many patients were excluded from the cited studies. 

The investigation of myocardial involvement under COVID-19 may be transferable to other diseases. In the future, subclinical myocardial dysfunction may be investigated in the context of other infectious diseases such as influenza, respiratory syncytial virus, Epstein–Barr virus and pneumococcal infections. The development of new and more pathogenic SARS-CoV-2 variants is also still conceivable.

## 5. Conclusions

STE with its main examination modalities, LV and RV longitudinal strain, is a good diagnostic tool for the detection of myocardial dysfunction, even in the presence of normal ejection fractions. It has shown its prognostic potential in acute COVID-19. Changes in both the right ventricular and left ventricular longitudinal strain were associated with higher mortality and morbidity in hospitalised COVID-19 patients. 

In survivors, the deformation parameters are mostly normal, although worse compared to healthy controls. Epidemiological studies are currently not available, so no statement can be made about the actual prevalence of myocardial dysfunction. Despite frequently small and, in part, contradictory studies, it can be said that the RV GLS seems to have a better tendency to recover after infection than the LV GLS. This is most likely due to different pathomechanisms with at least partially reversible changes in the pulmonary stromal bed for right ventricular dysfunction and scarring and persistent microvascular dysfunction as leading causes for left ventricular dysfunction. However, a reduction of the deformation parameters may have various reasons, and a conclusion regarding an exact pathomechanism is not possible in most cases.

## Figures and Tables

**Table 1:** Studies using speckle-tracking echocardiography with a focus on acute COVID-19.

Reference	Number of Patients	Subject	Main Finding
Stöbe S. et al. (August 2020) [17]	19	LV GLS, GCS, GRS Free wall RV LS	There are patterns of myocardial injury in hospitalised patients that differ from other cardiomyopathies, including viral myocarditis.
Li Y. et al. (November 2020) [19]	120	Free wall RV LS	Evaluating consecutive hospitalized patients, RV GLS was the best predictor of outcome and can help to identify patients at higher risk, with a cut-off of −23%.
Stockenhuber A. et al. (December 2020) [18]	30	Free wall RV LS	In hospitalised patients with elevated levels of troponin I, worsening free wall RV LS was associated with mortality.
Xie Y. et al. (January 2021) [22]	132	Four-chamber LV GLS Free wall RV LS	LV GLS (four chamber) and free wall RV LS were independent predictors of mortality and were useful tools in follow-up during convalescence of hospitalized patients.
Minhas A. et al. (June 2021) [24]	136	LV GLS MWE	In hospitalised patients, STE parameters were impaired in a significant proportion of patients. Worse values were associated with mortality and inflammatory biomarkers.
Shmueli H. et al. (January 2021) [23]	40	LV GLS	LV GLS was reduced in a high proportion of hospitalised patients. Impairment in LV GLS was correlated with elevated levels of troponin T and inflammatory biomarkers.
Bhatia H. et al. (March 2021) [26]	67	LV GLS	Despite normal LVEF, LV GLS was impaired in 91% of hospitalised patients, with improvement in those patients who survived to discharge.
Bleakley C. et al. (March 2021) [20]	90	Free wall RV LS	In patients in need of membrane oxygenation, free wall RV LS was not associated with cardiac biomarkers and could not show any additional benefit to other measurements of RV function.
Park J. (May 2021) [21]	48	LV GLS Free wall RV LS	In hospitalised patients, an LV GLS of greater than –13.8% was associated with a 5.15-fold increased risk of death. Free wall RV LS was not associated with that outcome.
D’Andrea A. et al. (May 2022) [28]	55 with signs for myocarditis in CMR	LV GLS	LV GLS in acute illness was associated with total scar burden in CMR and functional recovery at follow-up. Patients were initially hospitalized.
Ghantous E. et al. (January 2023) [25]	148 omicron propensity matched to wild type (alpha)	LV GLS RV GLS Free wall RV LS	All strain values were similar in patients with omicron variants and the wild type. Other hemodynamic and right ventricular parameters were worse in wild type patients. Patients included were in need of hospitalization.
Gonzalez F. et al. (January 2023) [27]	30	LA strain	In ICU patients, LA strain was more sensitive in detecting diastolic dysfunction than classical markers. It revealed protracted inflammatory states.

Abbreviations: Free wall RV LS: longitudinal strain of the right ventricular free wall; ICU: intensive care unit; LA, left atrial; LVEF: left ventricular ejection fraction; LV GLS: left ventricular global longitudinal strain; MWE: myocardial work efficiency; RV: right ventricular; RV GLS: right ventricular global longitudinal strain; STE: speckle-tracking echocardiography.

**Table 2:** Studies using speckle-tracking echocardiography with a focus on post-COVID. Note that the cohorts are very heterogeneous due to the individual inclusion criteria. This could not be presented here in full.

Reference	Number of Patients	Time after Infection	Subject	Main Finding
Ozer P. et al. (April 2021) [40]	79	133 ± 35 days	RV GLS Free wall RV LS	Impairment, especially in free wall RV LS, was correlated to disease severity, age, male sex, steroid treatment and presence of pneumonia in a chest CT scan.
Akkaya F. et al. (June 2021) [38]	105	3 months	RV GLS Free wall RV LS	In mild COVID-19, RV longitudinal strain was impaired and inversely correlated to inflammatory biomarkers and D-dimers.
Caiado L. et al. (July 2021) [33]	100	130 ± 70 days	LV GLS Segmental LS	LVEF and LV GLS were normal in post-COVID patients and comparable to healthy controls.
Mahajan S. et al. (September 2021) [41]	134	36 ± 5 days	LV GLS	Impaired LV GLS occurred in 29.9% of patients, with a significant correlation to initial disease severity.
Italia L. et al. (September 2021) [43]	123	85 days (IQR 70, 103)	LV GLS Free wall RV LS	The patients were divided into a group with and a group without troponin T elevation at follow-up as a marker of myocardial injury; LV GLS but not free wall RV LS was significant lower in the group with elevated troponin T.
Baruch G. et al. (September 2021) [37]	80	88 ± 33 days	Four-chamber LV GLS RV GLS	LV GLS was impaired in 33% of hospitalised patients, and RV GLS was impaired in 23%; significant improvement was observed in RV GLS but not in LV GLS (8% vs. 25% persistent deterioration, respectively).
Lassen M. et al. (October 2021) [29]	91	77 days (IQR 72, 92)	LV GLS RV GLS	During acute COVID-19, LV GLS and RV GLS were impaired compared to healthy controls. This impairment was fully reversible at follow-up in RV GLS but not in LV GLS.
Shimoni O. et al. (November 2021) [31]	184	57 days (IQR 27, 100)	LV GLS RV GLS Free wall RV LS	Strain values were reduced compared to those in controls but were not related to initial disease severity. Patients with an LV GLS of −20% or better had improved exercise capacities.
Luchian M. et al. (December 2021) [51]	66	1 year	LV GLS MWE	Patients with dyspnoea one year after COVID-19 had lower LV GLS and MWE.
Tryfou E. et al. (December 2021) [32]	100	33 ± 9 days	LV GLS Free wall RV LS	LV GLS was significantly impaired in all recovered COVID-19 patients, while free wall RV LS was significantly impaired only in formerly hospitalised patients.
Young K. et al. (June 2022) [36]	259	55 days (IQR 37, 92)	LV GLS RV GLS Free wall RV LS	On average, there were no clinically significant differences in STE pre- and post-COVID. Rare reductions were more common in patients with post-COVID symptoms.
Gherbesi E. et al. (September 2022) [50]	40	At least 3 months	LV GLS RV GLS Free wall RV LS	In young athletes with an asymptomatic or oligosymptomatic course of COVID-19, there was a reduction in LV GLS (but not RV GLS) compared to controls.
Garcia-Zamora S. et al. (August 2022) [35]	595	2 months (IQR 1, 3)	LV GLS Free wall RV LS	This registry study included all patients with STE following COVID-19. Overall, LV GLS and RV LS were normal, with abnormal values in 5.7% and 3.1% of patients, respectively.
Oikonomou E. et al. (September 2022) [30]	34	1 and 6 months after hospital discharge	LV GLS	One month after hospital discharge, LV GLS was impaired compared to healthy controls. After 6 months, LV GLS improved without reaching the level of the controls.
Baum P. et al. (November 2022) [44]	237	N/A	LV GLS	LV GLS was impaired in the subgroup of patients with worse fatigue and in patients with elevated troponin T. In most patients, LV GLS was normal.
ZeinElabdeen S. et al. (February 2023) [45]	63	4 to 12 weeks	LA strain	LA strain was impaired in symptomatic post-COVID patients. LA reservoir strain and LA stiffness were independent predictors of dyspnoea and exercise intolerance.

Abbreviations: Free wall RV LS: longitudinal strain of the right ventricular free wall; LV GLS: left ventricular global longitudinal strain; RV: right ventricular; RV GLS: right ventricular global longitudinal strain; STE: speckle tracking echocardiography.

## Data Availability

Not applicable.
